# Peritonsillar Ropivacaine Infiltration in Paediatric Tonsillectomy: A Randomised Control Trial

**DOI:** 10.22038/ijorl.2020.36577.2199

**Published:** 2020-07

**Authors:** Arvinder-Singh Sood, Pooja Pal, Gurupreet-Singh Gill

**Affiliations:** 1 *Department of Otorhinolaryngology, SGRD Institute of Medical Sciences and Research, Amritsar, Punjab, India – 143001.*

**Keywords:** Post operative analgesia, Ropivacaine, Tonsillectomy

## Abstract

**Introduction:**

Various efforts have been made to reduce post tonsillectomy pain in children. The present study was undertaken to evaluate the efficacy and safety of Ropivacaine in post-operative pain management in these children.

**Materials and Methods:**

This study is a Randomized control study on 140 patients between 4 - 12 years of age, in whom tonsillectomy was performed in a tertiary care centre between January 2017 to November 2018 using standard dissection and snare surgical technique. Postoperatively, patients were randomized into 2 groups of 70 patients each, receiving tonsillar fossa infiltration with 0.2% Ropivacaine or 0.9% normal saline respectively. Patients were assessed as per Wong Baker’s Faces Scoring System at 2 hours, 4 hours, 8 hours, 18 hours, 24 hours, and 48 hours postoperatively. All the results were analyzed by SPSS software. Chi- square test and Mann-Whitney test were used for assessment of level of significance, with P- value of less than 0.05 taken as significant.

**Results:**

Both the groups were comparable with respect to age and sex. At 48 hours, in study group, maximum number of patients 35 (50%) had Wong Baker score 0, while in control group, maximum number of patients 52 (74.3%) had Wong Baker score 4 (P< 0.01). The difference in the mean Deglutition time between both groups was significant (P< 0.01).

**Conclusion:**

Ropivacaine infiltration is a effective modality for post-tonsillectomy pain management in children, with minimal side-effects.

## Introduction

Acute inflammation of the tonsils can result in fever, tonsillar hypertrophy, dysphagia, reactionary lymphadenitis (1). 

Various efforts have been made to reduce postoperative pain including intra-operative anaesthetic medications, alteration of surgical technique, use of corticosteroids and intra-operative local anaesthetic injection. The probable mechanism of post-tonsillectomy pain is via the noxious stimulation of C-fibre afferents in the peritonsillar space.

It is hypothesized that sensory pathways responsible for the release of inflammatory factors may be inhibited by infiltration of a local anaesthetic agent, preventing the nociceptive impulses from reaching the spinal cord (2,3). The ideal agent used in infiltration should result in good analgesia reducing surgical morbidity, while having minimal side effects (4-6).

Ropivacaine is a new, synthetic, long-acting, amide-type local anaesthetic. Its high protein binding capacity (90-95%) results in a long duration of action of 6-8 hours, while its lower lipid solubility compared to lidocaine results in a delayed onset of action. Ropivacaine has less potential to cause serious cardiotoxic reactions compared to bupivacaine, making it a popular agent for epidural analgesia in gynecology and for sensory or motor blocks in surgery or orthopaedics. 

Although its role in the management of post tonsillectomy pain relief has been evaluated, there are conflicting reports, with studies involving less number of patients, and the results are controversial (7-9). 

Hence, the present study was undertaken to evaluate the efficacy of ropivacaine in the management of post tonsillectomy pain in children.

## Materials and Methods

The present study was a prospective, placebo-controlled, double blinded, randomized controlled study conducted in the Department of Otorhinolaryngology and Head Neck Surgery at Sri Guru Ram Das Institute of Medical Sciences and Research, Vallah, Amritsar after clearance from the Ethical committee (Ref. no.7664). This study was conducted over a period of two years, from January 2017 to November 2018 and included 140 consecutive patients between 4 - 12 years of age.

**Inclusion Criteria**

Patients with Chronic Adenotonsillitis or Bilateral Chronic Tonsillitis aged between 4-12 years.

- Scheduled to undergo Tonsillectomy with or without adenoidectomy

**Exclusion Criteria:**

1. Patients who have received any analgesic drug during the last 24 hours prior to surgery

2. Bleeding disorders

3. Any severe systemic illness

4. Unwilling or uncooperative patients

5. Asymmetric tonsillar enlargement

**Randomization and Allocation concealment**

Tonsillectomy was performed using a standard dissection surgical technique, and the inferior pole was clamped and cut using an Eve’s tonsillar snare. All procedures were performed under general anesthesia using a standardized protocol. Premedication was done using syrup midazolam 0.3-0.5 mg/kg, not exceeding 10ml. Following induction with intravenous propofol (2.5mg/kg), Injection Rocuronium (0.9mg/kg) and 100% oxygen, oral intubation was done. Anaesthesia was maintained with oxygen, nitrous oxide and isoflurane. Intravenous paracetamol at 15 mg/kg was given to all children irrespective of study or control group. Hemostasis was achieved using pressure with gauze pieces and judicious use of bipolar cautery. Following the procedure, the patients were randomized into 2 groups of 70 patients each by use of computer- generated random numbers into the following:

Group A (n=70): Tonsillar fossa infiltration with 3-5 ml of 0.2% Ropivacaine

Group B (n=70): Tonsillar fossa infiltration with 3-5 ml 0.9% normal saline

 The surgeon performing the procedure was handed an un-labeled syringe containing 3-5 ml of either Ropivacaine or normal saline depending on which group the patient belonged to. Extubation was performed after reversal of neuromuscular blocking agent.

Post-operative analgesia was divided into regular medications and rescue analgesia. Regular medication was administered according to the same protocol in both groups. 

All patients were given oral paracetamol and ibuprofen paediatric suspension in 2-3 divided doses. Rescue analgesia was given using a 10-15mg/kg/dose oral acetaminophen paediatric suspension in Wong Baker Score higher than 4. Time to first administration of acetaminophen, the total number of doses, and the total dosage administered was recorded. Postoperatively the patient was assessed as per Wong Baker’s Faces Scoring System in which the patient indicates to various facial smileys depending upon the severity of the pain (0=no hurt to 10= hurts worst). The scores were collected at 2 hours, 4 hours, 8 hours, 12 hours, 18 hours, 24 hours, and 48 hours postoperatively by an observer in the ward who was blinded to the solution infiltrated.

**Figure:**
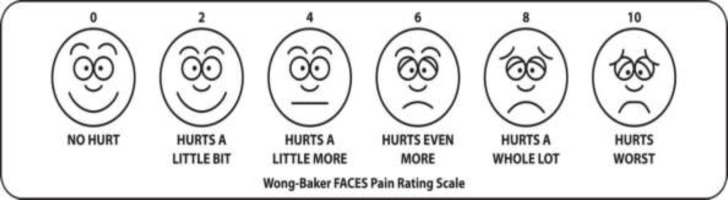


The observer also performed an objective assessment of pain by noting the deglutition time in seconds to swallow 100 ml of water from 4 hours post-operative onwards. 

Any side effects and complications if any, such as nausea or vomiting, fever and bleeding were documented. Data were collected in constructed proforma.

Statistical analysis 

All the results were analyzed by SPSS software version 23. Chi- square test and Mann-Whitney test were used for assessment of level of significance. P- value of less than 0.05 was taken as significant. 

## Results

The mean age of study group was 10.05 years and control group was 9.65 years. 

The study group comprised of 50 (71.4%) males and 20 (28.6%) females and control group had 47 (67.1%) males and 23 (32.9%) females (Table. 1).

**Table 1:** Distribution of subjects of the study group and control according to Age group

**Parameter**	**Mean age (years)**	**Standard deviation**
Study group	10.05	3.18
Control group	9.65	3.19

Table 2 shows distribution of subjects of the study group and the control group according to the Wong Baker score at 2 hours. Maximum number of subjects in study group .i.e. 40 (57.15%) had Wong Baker score 6. In control group, 67 (97.1%) subjects had Wong Baker score 8. Mann-Whitney test showed the difference to be highly significant (P< 0.01).

**Table 2:** Distribution of subjects of the study group and the control group according to the Wong Baker score at 2 hours

**Wong Baker score**	**Study group (n=70)**		**Control group (n=70)**		**Mann-Whitney U value**	**P- Value**
	**Number**	**Percentage**	**Number**	**Percentage**		
6	40	57.1	1	1.4	3890.0	0.00
8	30	42.9	68	97.1		
10	0	0	1	1.4		
Total	70	100	70	100		

 At 4 hours, 61 (87.1%) subjects in study group had Wong Baker score 6. Similarly, 65 (92.9%) subjects in control group had Wong Baker score 8. Mann-Whitney test showed that difference was statistically highly significant (P< 0.01) (Table.3).

**Table 3:** Distribution of subjects of the study group and the control group according to the Wong Baker score at 4 hours

**Wong Baker score**	**Study group (n=70)**		**Control group (n=70)**		**Mann-Whitney U value**	**P- Value**
	**Number**	**Percentage**	**Number**	**Percentage**		
4	1	1.4	0	0	4447.5	0.00
6	61	87.1	5	7.1		
8	8	11.4	65	92.9		
Total	70	100	70	100		

Table 4 shows distribution of subjects of the study group and the control group according to the Wong Baker score at 8 hours, maximum number of subjects 36 (51.4%) in study group had Wong Baker score 6 while 41 (58.6%) in control group had Wong Baker score 8. Mann-Whitney test showed that difference was statistically highly significant (P< 0.01).

**Table 4:** Distribution of subjects of the study group and the control group according to the Wong Baker score at8 hours

**Wong Baker score**	**Study group (n=70)**		**Control group (n=70)**		**Mann-Whitney U value**	**P- Value**
	**Number **	**Percentage **	**Number **	**Percentage **		
4	34	48.6	0	0	4378.0	0.00
6	36	51.4	29	41.4		
8	0	0	41	58.6		
Total	70	100	70	100		

At 12 hours, in the study group 58 (82.9%) subjects had Wong Baker score 4, while in the control group, 60 (85.7%) subjects had Wong Baker score 6. Mann-Whitney test showed that difference was statistically highly significant (P< 0.01) (Table.5).

**Table 5:** Distribution of subjects of the study group and the control group according to the Wong Baker score at 12 hours

**Wong Baker score**	**Study group (n=70)**		**Control group (n=70)**		**Mann-Whitney U value**	**P- Value**
	**Number**	**Percentage**	**Number**	**Percentage**		
2	1	1.4	0	0	4530.0	0.00
4	58	82.9	1	1.4		
6	11	15.7	60	85.7		
8	0	0	9	12.9		
Total	70	100	70	100		

Table 6 shows distribution of subjects of the study group and the control group according to the Wong Baker score at 18 hours. Maximum number of patients .i.e. 46 (65.7%) in study group had Wong Baker score 4, while 49 (70%) subjects in control group had Wong Baker score 6. Mann-Whitney test showed that difference was statistically highly significant (P< 0.01).

**Table 6:** Distribution of subjects of the study group and the control group according to the Wong Baker score at 18 hours

**Wong Baker score**	**Study group (n=70)**		**Control group (n=70)**		**Mann-Whitney U value**	**P- Value**
	**Number**	**Percentage**	**Number**	**Percentage**		
2	21	30	0	0	4358.0	0.00
4	46	65.7	18	25.7		
6	3	4.3	49	70.0		
8	0	0	3	4.3		
Total	70	100	70	100		

Table 7 shows that at 24 hours, maximum number of patients .i.e. 53 (75.7%) in study group had Wong Baker score 2, while in control group maximum number of patients .i.e. 41 (58.6%) had Wong Baker score 4. Mann-Whitney test showed that difference was statistically highly significant (P< 0.01).

**Table 7:** Distribution of subjects of the study group and the control group according to the Wong Baker score at 24 hours

**Wong Baker score**	**Study group (n=70)**		**Control group (n=70)**		Mann-Whitney U value	P- Value
	**Number **	**Percentage **	**Number **	**Percentage **		
0	2	2.9	0	0	4551.0	0.00
2	53	75.7	1	1.4		
4	15	21.4	41	58.6		
6	0	0	28	40.0		
Total	70	100	70	100		

At 48 hours, in the study group maximum number of patients .i.e. 35 (50%) had Wong Baker score 0, while in control group maximum number of patients .i.e. 52 (74.3%) had Wong Baker score 4. Mann-Whitney test showed that results were statistically highly significant (P< 0.0) (Table.8). 

**Table 8:** Distribution of subjects of the study group and the control group according to the Wong Baker score at 48 hours

**Wong Baker score**	**Study group (n=70)**		**Control group (n=70) **		**Mann-Whitney U value**	**P- Value**
	**Number **	**Percentage **	**Number **	**Percentage **		
0	35	50	1	1.4	4696.5	0.00
2	32	45.7	4	5.7		
4	3	4.3	52	74.3		
6	0	0	13	18.6		
Total	70	100	70	100		

Table 9 shows comparison of mean Deglutition time at different time intervals. The difference in the mean Deglutition time between the study and control groups at 4 hours, 8 hours, 12 hours, 18 hours, 24 hours and 48 hours was found to be statistically highly significant (P< 0.01).

**Table 9:** Comparison of mean Deglutition time at different time intervals

**Deglutition time**	**Study group (n=70)**		**Control group (n=70)**		Mann-Whitney U value	P- value
	**Mean time (seconds)**	**SD**	**Mean time (seconds)**	**SD**		
4 hours	158.85	39.36	220.85	30.96	4351.0	0.00
8 hours	134.71	32.91	196.14	28.55	4459.5	0.00
12 hours	108.14	31.77	176.42	27.24	4587.0	0.00
18 hours	87.14	29.74	149.28	29.05	4536.5	0.00
24 hours	54.85	27.58	124.00	26.12	4635.0	0.00
48 hours	35.50	16.10	96.57	20.63	4735.0	0.00

## Discussion

Pain after tonsillectomy and adenoidectomy is caused by inflammation, nerve irritation, and pharyngeal muscle spasms, resulting in the release of pro-inflammatory factors, chemokines, substance P, and vasoactive intestinal peptide. Sub-optimal postoperative pain relief can result in significant pain and postoperative nausea and vomiting (PONV), causing reduced oral intake, prolonged hospital stay, and may even require hospital readmission following discharge due to significant dehydration. This can be prevented by the judicious administration of an appropriate analgesic agent (10). In the present study, at two hours postoperative time, Wong baker score was 6 and 8 in 57.1% and 42.9% of the subjects of the study group (ropivacaine infiltration) respectively, and was 1.4% and 97.1% of the subjects of the control group (saline infiltration) respectively. While comparing the distribution of subjects of the study group and the control group according to the Wong Baker score at 2 hours, it was observed that subjects of the ropivacaine group had significant lower Wong Baker score in comparison to the subjects of the control group.

On comparing the study group and the control group at 4 hours postoperative time, it was observed that majority of the patients of the study group had Wong Baker score of 6 (61% patients), while majority of the patients of the control group had Wong Baker score of 8 (92.5%), the results of which were found to be statistically significant. 

A similar trend in the Wong Baker score was observed while comparing the study group and the control group at progressive postoperative time intervals. At 48 hours postoperatively, in 50% of patients of the study group, Wong Baker score of 0 was achieved, while among the subjects of the control group, only 1.4% of the subjects achieved the Wong baker score of 0.Similar results were reported by Goutham MK et al, who observed significantly better Wong Baker scale results postoperatively among subject of Ropivacaine group in comparison to the control group (11).

Our results were in concordance with the results obtained by Ju NY et al who reported similar findings. 

They found that combining 0.2% ropivacaine with dexamethasone resulted in superior analgesia in children undergoing tonsillectomy and adenoidectomy versus local anaesthetic infiltration with ropivacaine alone. This translated into decreased requirement for postoperative analgesics, improved oral intake and reduced postoperative nausea and vomiting (PONV), permitting earlier discharge (10).

The assessment of pain relief is a challenge in children, especially since they may sometimes not be forthcoming and able to express their emotions. The present studies employs two methods to evaluate the degree of postoperative analgesia, viz. the Wong Baker’s Faces Scoring System and the eglutition time for 100ml of water. 

This is in contrast to majority of previous studies which employ only the visual analogue scale (VAS) or dynamic pain assessments (e.g. when drinking water or opening the jaw) to assess the degree of postoperative pain relief. However, some authors admit that the VAS scale may often be confusing for children to use. 

## Conclusion

Ropivacaine is an ideal agent for local infiltration for postoperative analgesia following tonsillectomy in children in terms of both safety and efficacy.
